# Preferential and persistent impact of acute HIV-1 infection on CD4^+^ iNKT cells in colonic mucosa

**DOI:** 10.1073/pnas.2104721118

**Published:** 2021-11-09

**Authors:** Dominic Paquin-Proulx, Kerri G. Lal, Yuwadee Phuang-Ngern, Matthew Creegan, Andrey Tokarev, Suchada Suhkumvittaya, Aljawharah Alrubayyi, Eugène Kroon, Suteeraporn Pinyakorn, Bonnie M. Slike, Diane L. Bolton, Shelly J. Krebs, Leigh Anne Eller, Chayada Sajjaweerawan, Amélie Pagliuzza, Nicolas Chomont, Rungsun Rerknimitr, Nitiya Chomchey, Nittaya Phanuphak, Mark S. de Souza, Nelson L. Michael, Merlin L. Robb, Jintanat Ananworanich, Johan K. Sandberg, Michael A. Eller, Alexandra Schuetz, RV217, RV254/SEARCH010, RV304/SEARCH013, Study Groups

**Affiliations:** ^a^US Military HIV Research Program, Walter Reed Army Institute of Research, Silver Spring, MD 20910;; ^b^Henry M. Jackson Foundation for the Advancement of Military Medicine, Inc., Bethesda, MD 20817;; ^c^Center for Infectious Medicine, Department of Medicine Huddinge, Karolinska Institutet, Karolinska University Hospital Huddinge, Stockholm 141 52, Sweden;; ^d^Department of Retrovirology, Armed Forces Research Institute of Medical Sciences, Bangkok 10400, Thailand;; ^e^Institute of HIV Research and Innovation, Bangkok 10330, Thailand;; ^f^Centre de Recherche du Centre Hospitalier de l'Université de Montréal, Université de Montréal, Montréal, Québec H2X 0A9, Canada;; ^g^Faculty of Medicine, Chulalongkorn University, Bangkok 10330, Thailand;; ^h^AIDS Research Center, National Institute of Infectious Diseases, Tokyo 162-8640, Japan;; ^i^Center for Infectious Disease Research, Walter Reed Army Institute of Research, Silver Spring, MD 20910;; ^j^Department of Global Health, Amsterdam Medical Center, University of Amsterdam, Amsterdam 1105, The Netherlands;; ^k^Vaccine Research Program, Division of AIDS, National Institute of Allergy and Infectious Diseases, NIH, Bethesda, MD 20892

**Keywords:** HIV-1, iNKT cells, ART, gut, immune activation

## Abstract

Acute HIV-1 infection (AHI) results in the widespread depletion of CD4^+^ T cells in peripheral blood and gut mucosal tissue. However, the impact on the predominantly CD4^+^ immunoregulatory invariant natural killer T (iNKT) cells during AHI remains unknown. Here, iNKT cells from peripheral blood and colonic mucosa were investigated during treated and untreated AHI. iNKT cells in blood were activated and rapidly depleted in untreated AHI. At the time of peak HIV-1 viral load, these cells showed the elevated expression of cell death–associated transcripts compared to preinfection. Residual peripheral iNKT cells suffered a diminished responsiveness to in vitro stimulation early into chronic infection. Additionally, HIV-1 DNA, as well as spliced and unspliced viral RNA, were detected in iNKT cells isolated from blood, indicating the active infection of these cells in vivo. The loss of iNKT cells occurred from Fiebig stage III in the colonic mucosa, and these cells were not restored to normal levels after initiation of ART during AHI. CD4^+^ iNKT cells were depleted faster and more profoundly than conventional CD4^+^ T cells, and the preferential infection of CD4^+^ iNKT cells over conventional CD4^+^ T cells was confirmed by in vitro infection experiments. In vitro data also provided evidence of latent infection in iNKT cells. Strikingly, preinfection levels of peripheral blood CD4^+^ iNKT cells correlated directly with the peak HIV-1 load. These findings support a model in which iNKT cells are early targets for HIV-1 infection, driving their rapid loss from circulation and colonic mucosa.

## Significance

Evidence suggests that HIV-1 disease progression is determined in the early stages of infection. Here, preinfection invariant natural killer T (iNKT) cell levels were predictive of the peak viral load during acute HIV-1 infection (AHI). Furthermore, iNKT cells were preferentially lost in AHI. This was particularly striking in the colonic mucosa, where iNKT cells were depleted more profoundly than conventional CD4^+^ T cells. The initiation of antiretroviral therapy during AHI-prevented iNKT cell dysregulation in peripheral blood but not in the colonic mucosa. Overall, our results support a model in which iNKT cells are early and preferential targets for HIV-1 infection during AHI.

Invariant natural killer T (iNKT) cells are unconventional, CD1d-restricted T cells with innate-like properties (1, 2). Following the recognition of glycolipid antigens, iNKT cells respond with a broad range of effector and regulatory functions (3, 4). In humans, iNKT cells invariably use the Vα24-Jα18 T cell receptor chain preferentially paired to the Vβ11 chain and can be divided into CD4^+^ and CD4^−^ subsets. The CD4^−^ subset is biased toward a Th1 profile and cytotoxic function, and the CD4^+^ subset is associated with a Th2 profile and can provide help to other immune cell subsets, such as B cells, macrophages, and NK cells (5–7). Murine studies have shown that iNKT cells can contribute to the clearance of viral infections (8, 9). They have been demonstrated to directly recognize hepatitis B virus-infected cells by their T cell receptor (TCR) (10) and to play an important role in the initiation of antiviral B cell responses (11, 12).

In HIV-1 infection, iNKT cells are depleted (13–15), and the remaining cells produce less cytokines following in vitro stimulation (16, 17). Additionally, iNKT cells are susceptible to HIV-1 infection in vitro (14, 18), but this has not been corroborated ex vivo. The initiation of antiretroviral treatment limits the decline of iNKT cells and improves functionality (19, 20). Interestingly, individuals who do not progress to AIDS, or individuals who control viremia in the absence of ART, maintain a normal frequency of iNKT cells (21–23). A direct role for iNKT cells in the control of HIV-1 infection is supported by the up-regulation of endogenous CD1d-presented glycolipid antigens by HIV-infected dendritic cells (24). Furthermore, colonic iNKT cells are thought to be associated with the control of HIV-1–induced inflammation (22, 25). Taken together, these observations suggest that iNKT cells can influence the progression of HIV-1 disease. However, it remains unknown how iNKT cells respond during acute HIV-1 infection (AHI), a period which is associated with inflammation, damage to the gastrointestinal (GI) tract, and the loss of memory CD4^+^ T cells (26). The early initiation of antiretroviral therapy (ART) during the acute phase of infection limits HIV-1 reservoir seeding and immune activation (27–29) and preserves important subsets of CD4^+^ T cells in the gut mucosa (30, 31), whereas the fate of iNKT cells has not been studied. Therefore, the mechanism and timing of HIV-induced iNKT cell dysregulation in the blood and GI tract are still not understood.

In this study, we investigated iNKT frequency, phenotype, and function longitudinally in individuals prior to infection and in AHI with or without ART. Our results suggest that the loss of colonic and peripheral blood CD4^+^ iNKT cells occurs as early as AHI. Furthermore, in the colonic mucosa, CD4^+^ iNKT cell depletion was more profound than for conventional CD4^+^ T cells. The limited reconstitution of colonic CD4^+^ iNKT cells occurred during the 2 y following ART initiation. In addition, we found evidence suggesting that CD4^+^ iNKT cells may contribute to HIV-1 viral reservoirs. Overall, our findings support a model in which CD4^+^ iNKT cells may be more susceptible to HIV-1 infection than conventional CD4^+^ T cells during AHI.

## Results

### Peripheral Blood CD4^+^ iNKT Cells Are Lost during AHI.

**Fig. 1.**
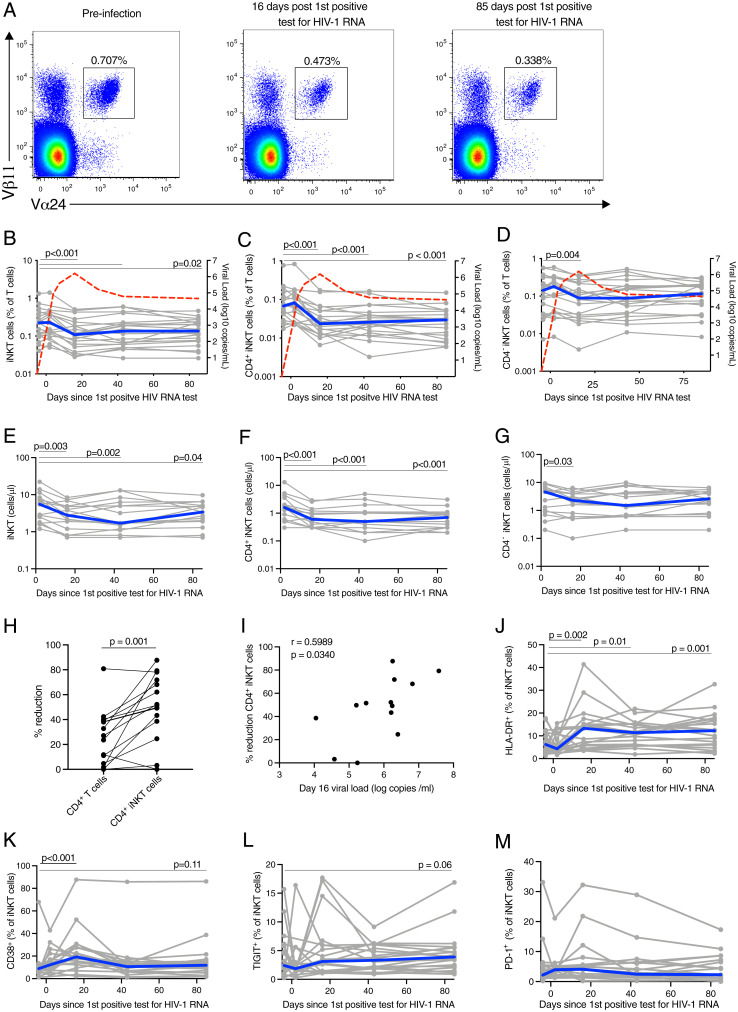
Peripheral blood CD4^+^ iNKT are reduced early in untreated AHI. (*A*) Representative flow plots showing the frequency of peripheral blood iNKT in acute, untreated HIV-1 infection. Frequency of peripheral blood iNKT (*B*), CD4^+^ iNKT (*C*), and CD4^−^ iNKT (*D*) cells in acute, untreated HIV-1 infection. Individual subjects are shown in gray and the median in blue. The red line represents the median VL. Absolute cell count of peripheral blood iNKT (*E*), CD4^+^ iNKT (*F*), and CD4^−^ iNKT (*G*) cells in acute, untreated HIV-1 infection. (*H*) Percentage of absolute cell count reduction for conventional CD4^+^ T cells and CD4^+^ iNKT cells. (*I*) Association between the VL at day 16 after the first HIV RNA–positive test and the percentage of CD4^+^ iNKT cell reduction. Expression of HLA-DR (*J*), CD38 (*K*), TIGIT (*L*), and PD-1 (*M*) by peripheral blood iNKT cells in acute, untreated HIV-1 infection. Time points sampled are indicated by the circles. *n* = 20 for all plots, except for *E*, *F*, *G,* and *H* in which *n* = 15 and *I* in which *n* =13.

The frequency and phenotype of peripheral blood iNKT cells were investigated longitudinally prior to infection and during untreated AHI in 20 participants from the HIV ECHO (RV217) acute infection cohort from East Africa and Thailand (32) (Table 1). Peripheral blood mononuclear cells (PBMCs) collected preinfection, and at early infection, peak viral load (VL), VL set point, and early chronic infection corresponding to a median of 2, 16, 43, and 85 d after the first HIV-1 RNA–positive test, respectively, were analyzed. A reduced frequency of iNKT cells was evident already at peak VL (*P* < 0.001; Fig. 1 *A* and *B*), compared to preinfection, and remained diminished into early chronic infection (*P* = 0.02). This pattern was more pronounced in the CD4^+^ iNKT cell subset (*P* < 0.001 at all time points; Fig. 1*C*), while the frequency of CD4^−^ iNKT cells was reduced at the peak VL time point (*P* = 0.004) but subsequently returned to preinfection levels (Fig. 1*D*). Longitudinal absolute cell counts starting from day 2 after the first HIV RNA–positive test were available in a subset of 15 participants, and comparable results were obtained (Fig.1 *E*–*G*). The loss between days 2 and 16 after the first HIV RNA–positive test was significantly worse for CD4^+^ iNKT cells compared to conventional CD4^+^ T cells (Fig. 1*H*). The percentage of CD4^+^ iNKT cell reduction was associated with VL at day 16 after the first HIV RNA–positive test (Fig. 1*I*). We evaluated cell surface markers of activation (HLA-DR and CD38) and exhaustion (TIGIT and PD-1) on iNKT cells (*SI Appendix*, Fig. S1). The expression of HLA-DR and CD38 were significantly elevated at peak VL compared to preinfection (*P* = 0.002 and *P* < 0.001, respectively) (Fig. 1 *J* and *K*). HLA-DR expression remained elevated at VL set point and early into chronic infection (*P* = 0.01 and *P* = 0.001, respectively), while CD38 expression returned to preinfection levels. No significant change in the expression of TIGIT and PD-1 was observed at any time after HIV-1 infection compared to preinfection, although a trend toward increased TIGIT was detected at early chronic infection (*P* = 0.06) (Fig. 1 *L* and *M*). This was in contrast to the dynamics of conventional CD4^+^ T cells that had a sustained increase in CD38 levels starting from peak VL and increased PD-1 expression at days 16 and 85 after the first HIV-1 RNA–positive test (*SI Appendix*, Fig. S2). There was no difference in the activation of CD4^+^ and CD4^−^ iNKT cells in a subset of study participants for which a sufficient number of events were recorded for both subsets (*SI Appendix*, Fig. S3). We hypothesized that the increased activation of iNKT cells might be linked to viral replication, the release of inflammatory cytokines, or microbial translocation; hence, the levels of soluble CD14 (sCD14), IL-12, and IL-6 in plasma during AHI were assessed (*SI Appendix*, Fig. S4). Levels of HLA-DR and CD38 on iNKT cells at peak VL showed no significant associations with VL, sCD14, IL-12, or IL-6 levels at the same time point (*SI Appendix*, Fig. S4). It should, however, be noted that it remains possible that iNKT cell activation may be associated with other soluble factors that were not measured in this study. These results suggest that iNKT cells are affected during untreated AHI starting from peak VL.

**Table 1.** Acute untreated (RV217) subjects’ demographics

Characteristics	Acute HIV infected (*n* = 22)	HIV uninfected (*n* = 20)
Median age [years]	23 (18, 35)*	25 (18, 45)*
Gender Male:Female:MtF	8:9:5	2:17:1
Country, *n* (%)		
Uganda	6 (27.4)	5 (20)
Tanzania	3 (13.6)	5 (20)
Kenya	2 (9)	5 (20)
Thailand	11 (50)	5 (20)
Median CD4^+^ T cell nadir (cells/μL)	483 (286, 866)*	NA
Median time to peak VL (d)	14 (6, 19)*	NA
Median peak VL (log_10_ copies/mL)	6.68 (5.49, 7.94)*	NA
Median set point VL (log_10_ copies/mL)	4.46 (3.52, 5.96)*	NA

*range

CD4+ T cell nadir = minimum CD4+ T cell count prior to day 80; set point VL = average of all measured VL between day 80 and day 365, in the absence of treatment (required at least two measurements); MtF = Male to female transgender and NA: Not Applicable.

**Fig. 2.**
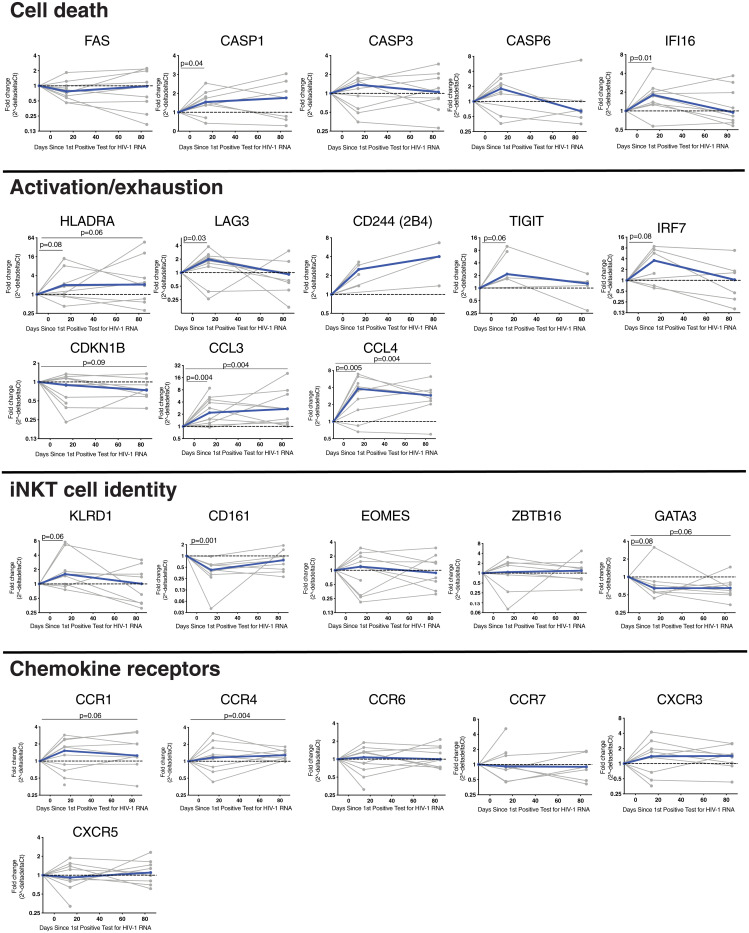
Change in iNKT cell gene expression during AHI. Bulk iNKT cells from 10 subjects from preinfection, peak VL (corresponding to 16 d after the first positive HIV-1 RNA–positive test), and early chronic infection (corresponding to 85 d after the first positive HIV-1 RNA–positive test) were sorted. RNA was extracted, and complementary DNA was generated using target-specific primers. Gene expression was quantified by qPCR and compared to preinfection levels. Individual subjects are shown in gray and the median in blue. Time points sampled are indicated by the circles.

Next, targeted gene expression profiles were studied in sorted iNKT cells from a subset of 10 individuals during AHI and at early chronic infection to characterize the expression of transcripts associated with cell death, activation, exhaustion, lineage, and chemokine receptor expression. The expression of *KLRB1* and *ZBTB16* were increased in sorted iNKT cells compared to conventional CD4^+^ T cells (*SI Appendix*, Fig. S5). The expression of several genes associated with immune activation and exhaustion were elevated by a median of twofold or more at peak VL, compared to preinfection, with many returning to preinfection levels by the early chronic infection time point (Fig. 2). However, the expression of HLA-DR, 2B4, CCL3, and CCL4 genes remained elevated in early chronic infection. *IRF7* and *CCL4* showed the strongest up-regulation during AHI (median 3.6-fold increase compared to preinfection). Transcripts for caspases 1 (median 1.5-fold), 3 (median 1.4-fold), and 6 (median 1.8-fold), all proteases involved in programmed cell death, as well as *IFI16* (median 1.8-fold), a DNA sensor involved in cell death and associated with HIV-1 infection (33), were also increased by peak VL, but only levels of transcripts for caspase 1 and IFI16 reached statistical significance. No changes were detected for the gene expression of *ZBTB16* [encoding the transcription factor PLZF, which is required for iNKT cell development (34, 35)]. Conversely, expression levels of *GATA3*, a gene important for iNKT cell development and function (36), were reduced at peak VL and remained reduced into the early chronic infection stage (0.6-fold at both time points). The expression of chemokine receptor genes were mostly unchanged during AHI with the exception of *CCR4*, which increased 1.3-fold during early chronic infection compared to preinfection. Overall, these results show the modulation of the iNKT cell transcriptional profile at peak VL, indicating the activation of iNKT cells.

### Preinfection Levels of Peripheral Blood CD4^+^ iNKT Cells Are Associated with Peak Viral Load.

**Fig. 3.**
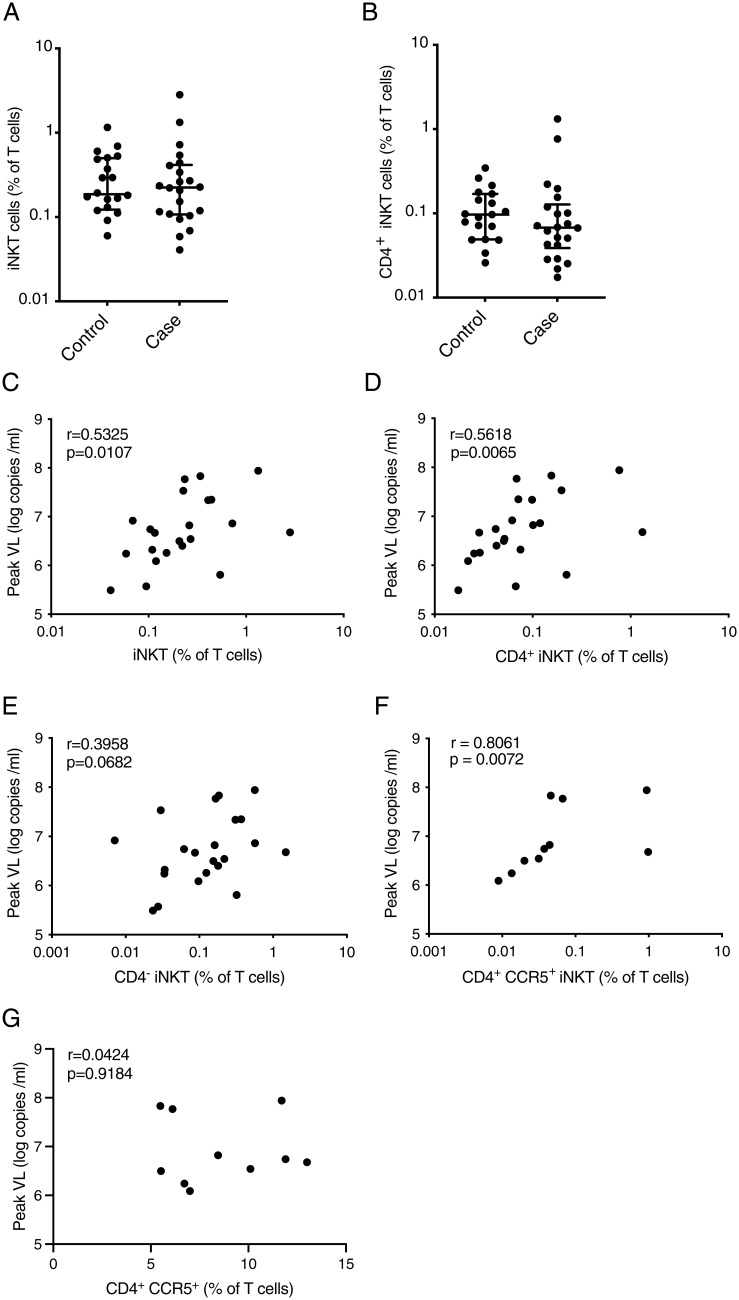
Preinfection levels of CD4^+^ iNKT cells are associated with the peak VL. Frequency of peripheral blood iNKT (*A*) and CD4^+^ iNKT cells (*B*) for individuals enrolled in RV217 that did not become HIV infected (*n* = 20) or became HIV infected (*n* = 22). The lines and whiskers represent the median and interquartile range, respectively. Associations between peripheral blood iNKT (*C*), CD4^+^ iNKT (*D*), and CD4^−^ iNKT cells (*E*) preinfection frequencies and peak VL (*n* = 22). Associations between peripheral blood CD4^+^ CCR5^+^ iNKT cells (*n* = 10) (*F*) or conventional CD4^+^ CCR5^+^ T cells (*n* = 10) (*G*) frequency and peak VL.

Next, we investigated characteristics of iNKT cells for associations with HIV acquisition or disease progression. To this end, we compared the frequency of total iNKT cells and the CD4^+^ iNKT cell subset at enrollment between 22 individuals that became HIV-1 infected and 20 individuals that did not become HIV-1 infected during the course of the study and found no difference between the groups (Fig. 3 *A* and *B*). Of note, there was also no difference in iNKT and CD4^+^ iNKT cell frequencies between African and Thai study participants (*SI Appendix*, Fig. S6 *A* and *B*). However, the frequency of iNKT cells preinfection correlated with peak VL (rho = 0.5325 and *P* = 0.0107; Fig. 3*C*), and this association was stronger for the CD4^+^ subset (rho = 0.5618 and *P* = 0.0065; Fig. 3*D*) compared to CD4^−^ iNKT cells (rho = 0.3958 and *P* = 0.0682; Fig. 3*E*). For 10 individuals, CCR5 expression was measured preinfection, and the frequency of blood CCR5^+^ CD4^+^ iNKT cells, but not conventional CCR5^+^ CD4^+^ T cells, was associated with peak VL (Fig. 3 *F* and *G*). CCR5^+^ CD4^+^ iNKT cells were not correlated with CD4^+^ T cell count nadir, viral set point, or the expression of activation markers by iNKT cells. Taken together, these data suggest that CD4^+^ CCR5^+^ iNKT cells are associated with the magnitude of viral replication at the earliest stages of HIV infection.

### ART Initiation in Fiebig Stages I or II Is Associated with the Maintenance of Circulating CD4^+^ iNKT Cells and Function.

**Fig. 4.**
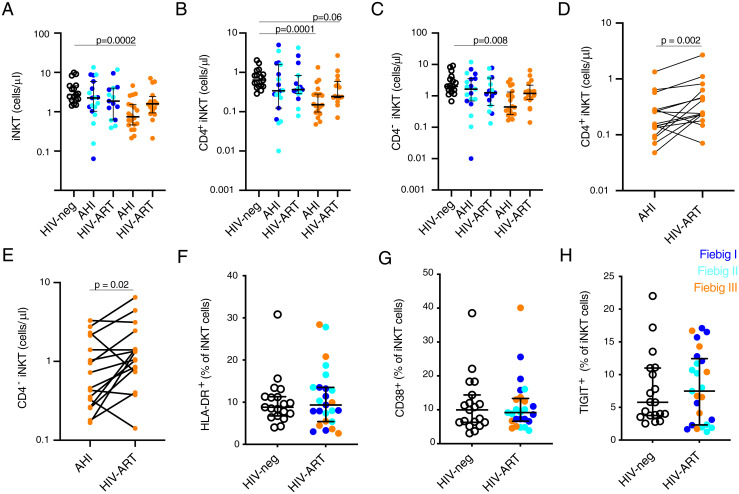
Initiation of ART in AHI prevents the loss of peripheral blood CD4^+^ iNKT cells. Absolute cell count of peripheral blood iNKT (*A*), CD4^+^ iNKT (*B*), CD4^−^ iNKT (*C*) cells in HIV-uninfected subjects (HIV-neg black, *n* = 17), Fiebig I (dark blue), Fiebig II (light blue), and Fiebig III (orange) HIV-infected individuals before (HIV acute, *n* = 39) and after 24 mo of ART (*n* = 31). Paired pre- and post-ART absolute CD4^+^ (*D*) and CD4^−^ (*E*) iNKT cell count in study participants that initiated ART in Fiebig stage III (*n* = 16). Expression of HLA-DR (*F*), CD38 (*G*), and TIGIT (*H*) by peripheral blood iNKT cells in subjects that initiated ART in AHI (HIV uninfected *n* =19, Fiebig 1 *n* = 9, Fiebig II *n* = 9, and Fiebig III *n* = 7) 6 mo after ART. The lines and whiskers represent the median and interquartile range, respectively.

To determine if early ART treatment can prevent the loss or limit the activation of peripheral blood iNKT cells, we analyzed PBMC samples from the RV254/SEARCH010 AHI cohort from Thailand (37). Age-, gender- and risk group–matched, HIV-1–uninfected Thai individuals were used as a control group (Table 2). During AHI, the absolute count of total iNKT, CD4^+^, and CD4^−^ iNKT cells were reduced beginning in Fiebig stage III (Fig. 4 *A*–*C*). Following 2 y of suppressive ART, initiated during AHI, the absolute counts were similar to those of HIV-uninfected individuals, although there was trend of lower CD4^+^ iNKT cells in participants that initiated ART during Fiebig stage III (Fig. 4 *A* and *B*). CD4^+^ and CD4^−^ iNKT cell count were increased following 2 y of ART initiated during Fiebig stage III in a subset of study participants with matched samples from both time points (Fig. 4 *D* and *E*). Similarly, the expression of HLA-DR, CD38, and TIGIT by iNKT cells did not differ between HIV-uninfected subjects and HIV-1–infected individuals that initiated ART during AHI (Fig. 4 *F*–*H*). These results suggest that the initiation of ART during AHI can mitigate the HIV-1–associated activation and loss of peripheral blood iNKT cells.

**Table 2.** Clinical, immunological, and virological characteristics and demographics of RV254/SEARCH 010 and RV304/SEARCH 013 study participants

Characteristics	Acute HIV infected at time of diagnosis (*n* = 39)	Acute HIV infected post-ART initiation (*n* = 31)	HIV uninfected (*n* = 28)
Median age at visit (y)	28 (19–48)*	30 (21–48)*	32 (20–43)*
Gender Male:Female:MtF	36:3:0	28:3:0	18:6:4
Risk behavior, *n* (%)			
MSM	32 (82.0)	25 (80.7)	14 (50)
Bisexual male	3 (7.7)	2 (6.4)	—
Heterosexual male	1 (2.6)	1 (3.2)	4 (14.3)
Heterosexual female	3 (7.7)	3 (9.7)	6 (21.4)
MtF	—	—	4 (14.3)
Fiebig Stage, *n*			
I/II	19 (48.7)	15 (48.4)	NA
III	20 (51.3)	16 (51.6)	NA
Mean (SD) duration of HIV (d)	16 (7.1)	15 (7.0)	NA
Mean (SD) time to ART initiation (d)		5 (1.4)	NA
Median duration of ART (wk)	NA	96 (96–96)*	NA
Median plasma HIV RNA (log_10_ copies/mL)	5.5 (2.8–7.7)*	1.7 (1.3–1.8)*	NA
Median sigmoid colon HIV RNA (log_10_ copies/mg tissue)	2.7 (1.3–6.5)* (*n* = 36)	1.7 (1.7–1.8)* (*n* = 14)	NA
Median CD4^+^ T cell count (cell/mm^3)^	428 (132–1,127)*	618 (451–1,200)*	1,054 (738–2,059)* (*n* = 17)

*range

MSM: Men who have sex with men; MtF: male to female; Fiebig I—positive HIV RNA, negative p24 antigen, and negative third generation enzyme immunoassay (EIA); Fiebig II—positive HIV RNA, positive p24 antigen, and negative third generation EIA; Fiebig III—positive HIV RNA, positive p24 antigen, positive third generation EIA, and negative Western blot; and NA: Not Applicable.

**Fig. 5.**
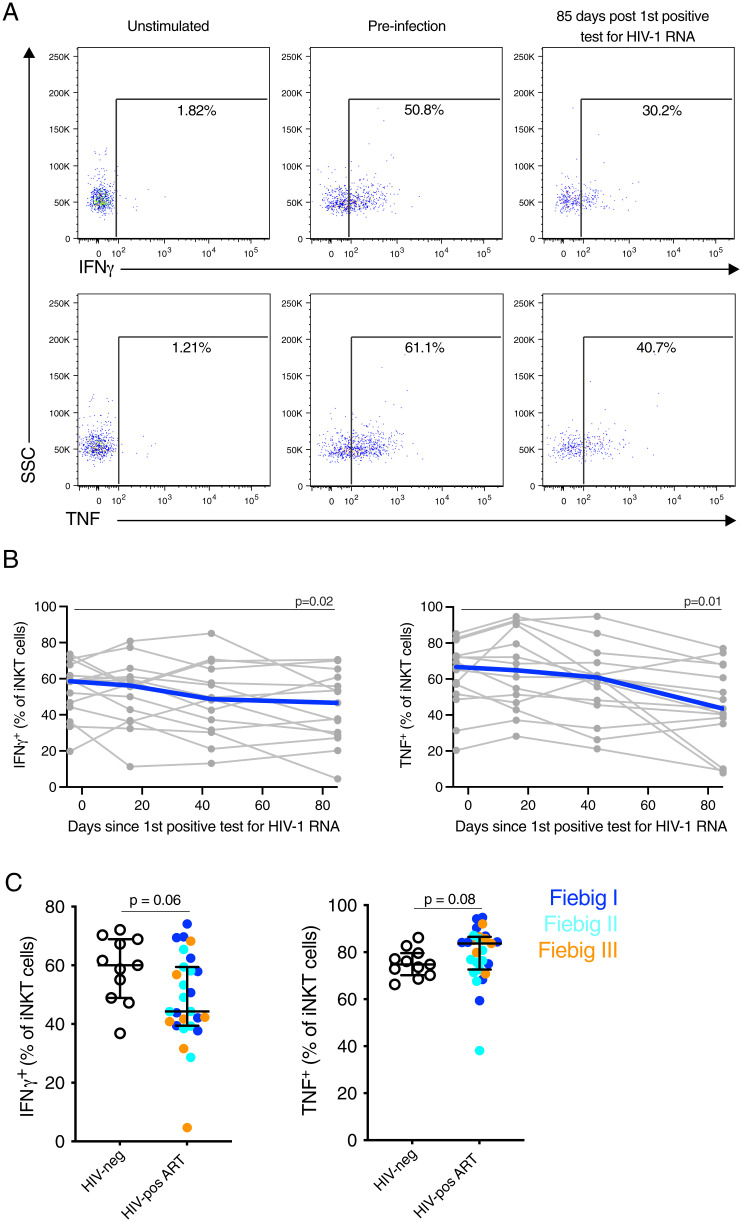
Initiation of ART in AHI prevents the reduction of cytokine production by iNKT cells. PBMCs were stimulated with PMA and Ionomycin for 6 h, and the production of IFN-γ and TNF by iNKT cell was evaluated by flow cytometry. (*A*) Representative flow plots showing production of IFNγ and TNF by iNKT cells pre- and 85 d post-first positive HIV-1 RNA positive test (early chronic) in untreated subjects. (*B*) IFN-γ (*Left*) and TNF (*Right*) production by iNKT cells before and in early chronic HIV-1 infection in untreated subjects (*n* = 15). Individual subjects are shown in gray and the median in blue. Time points sampled are indicated by the circles. (*C*) IFN-γ (*Left*) and TNF (*Right*) production by iNKT cells in subjects that initiated ART in AHI (HIV uninfected *n* = 11 [HIV-neg], Fiebig 1 *n* = 10, Fiebig II *n* = 10, and Fiebig III *n* = 7 [HIV-pos]) 6 mo after ART.

Studies of chronic HIV-1 infection have reported a reduced potential of peripheral blood iNKT cells to produce cytokines following in vitro stimulation (16, 17, 38). To assess the impact of acute infection on iNKT cell function, we evaluated the production of IFN-γ and TNF by peripheral blood iNKT cells from untreated AHI after stimulation with phorbol 12-myristate 13-acetate (PMA) and ionomycin (Fig. 5*A*). No changes in iNKT cell functional response were observed at peak VL or VL set point (median day 16 and 43 after the first positive HIV RNA test, respectively) compared to preinfection levels (Fig. 5*B*). However, a significant decrease in both IFN-γ (*P* = 0.02) and TNF (*P* = 0.01) production by iNKT cells was observed at the early chronic time point (median day 85 after the first positive HIV RNA test) compared to preinfection levels (Fig. 5*B*). Next, we investigated if starting ART during AHI may alleviate the functional decline of iNKT cells observed in untreated, chronic HIV-1 infection. Indeed, iNKT cells from subjects treated in AHI for 6 mo maintained a similar production of IFN-γ and TNF after in vitro stimulation compared to HIV-1–uninfected individuals, although there was a trend for reduced IFN-γ production in individuals treated in Fiebig stage III (*P* = 0.06) (Fig. 5*C*). Similar results were obtained for conventional CD4^+^ T cells (*SI Appendix*, Fig. S7).

### ART Initiation during Fiebig Stage III Does Not Prevent the Loss of Colonic CD4^+^ iNKT Cells.

**Fig. 6.**
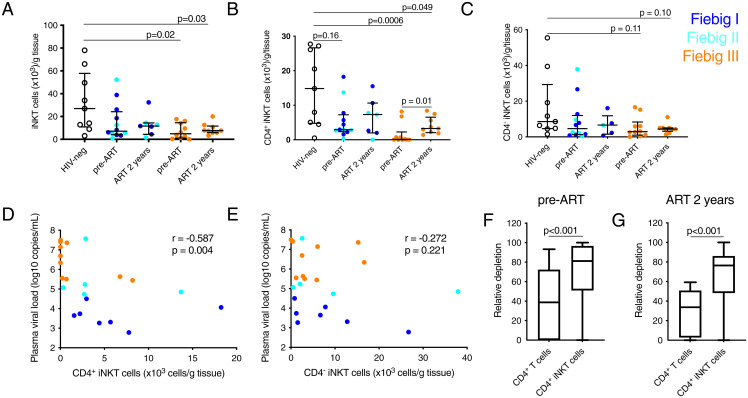
Colonic CD4^+^ iNKT cells are preferentially depleted in AHI and show limited recovery after ART. Absolute number of colonic iNKT cells (*A*), CD4^+^ iNKT cells (*B*), and CD4^−^ iNKT cells (*C*) in HIV-uninfected subjects (HIV-neg black, *n* = 9), Fiebig I (dark blue) Fiebig II (light blue), and Fiebig III (orange) HIV-infected individuals before ART (*n* = 22) and after 2 y of ART (*n* = 16). The lines and whiskers represent the median and interquartile range, respectively. Associations between plasma VL and absolute numbers of colonic CD4^+^ (*D*) and CD4^−^ (*E*) iNKT cells. The relative depletion of CD4^+^ T cells and CD4^+^ iNKT cells before ART (*F*) and after 2 y of ART (*G*) were calculated by comparing values for each HIV-infected individual to the median value for the HIV-uninfected group. The whiskers represent the minimum to maximum and the box represent the 25% percentile, median, and 75% percentile.

The GI tract is a major site of HIV replication and immunopathogenesis (26, 39, 40). Interestingly, colonic iNKT cells may mitigate HIV-associated inflammation (22, 25). Therefore, we next investigated the frequency of colonic iNKT cells at AHI diagnosis and 2 y after immediate ART initiation. The absolute number of iNKT cells per gram of tissue was reduced in AHI compared to uninfected subjects starting from Fiebig stage III (*P* = 0.02), with the preferential depletion of the CD4^+^ subset (*P* = 0.0006; Fig. 6 *A*–*C*). Colonic CD4^+^ iNKT cells recovered after 2 y of ART compared to AHI in participants that initiated ART in Fiebig stage III (*P* = 0.01), but the levels remained lower than in HIV-uninfected individuals (*P* = 0.049). There was a significant inverse correlation between the plasma VL and the concentration of colonic CD4^+^ iNKT cells (rho = −0.587 and *P* = 0.004) but not of CD4^−^ iNKT cells (rho = −0.272 and *P* = 0.221) (Fig. 6 *D* and *E*). Furthermore, the relative depletion of colonic CD4^+^ iNKT cells was significantly higher than that of conventional CD4^+^ T cells, when comparing HIV-1–infected to uninfected individuals, during both AHI and after 2 y of ART (*P* < 0.001; Fig. 6 *F* and *G*). This suggests a pattern of preferential loss of colonic CD4^+^ iNKT cells during AHI and a relatively limited recovery compared to conventional CD4^+^ T cells in response to ART.

We investigated the loss of colonic CD4^+^ iNKT cells in relation to indices of immune activation. An inverse association was observed between colonic CD4^+^ iNKT cells and plasma levels of IP-10 during AHI (*SI Appendix*, Fig. S8*A*). In addition, colonic CD4^+^ iNKT cell frequencies were inversely associated with the levels of colonic regulatory T cells (*SI Appendix*, Fig. S8*B*).

### iNKT Cells Express Markers Associated with Susceptibility to HIV Infection.

Next, the surface expression of HIV-1 coreceptors was evaluated to assess the potential susceptibility of CD4^+^ iNKT cells to infection. Peripheral blood CD4^+^ iNKT cells expressed higher levels of CCR5 and α4β7 than conventional CD4^+^ T cells (*SI Appendix*, Fig. S9 *A*–*D*). Similarly, CCR5 expression was higher on colonic CD4^+^ iNKT cells than conventional CD4^+^ T cells, both in terms of the percentage of expression and level of expression on the positive cells (*SI Appendix*, Fig. S9 *E* and *F*). To confirm their susceptibility to HIV-1 infection, peripheral blood conventional T cells and iNKT cells were cultured in vitro with HIV-1 BaL, after which p24 levels were measured as a marker of productive infection (*SI Appendix*, Fig. S10*A*). The percentage of productively infected cells was higher for iNKT cells compared to conventional CD4^+^ T cells for all donors (*P* = 0.03; *SI Appendix*, Fig. S10*B*). These results suggest that CD4^+^ iNKT cells are more susceptible to infection with HIV-1 than conventional CD4^+^ T cells.

### iNKT Cells Are Infected In Vivo and Express Markers Associated with the HIV Reservoir.

**Fig. 7.**
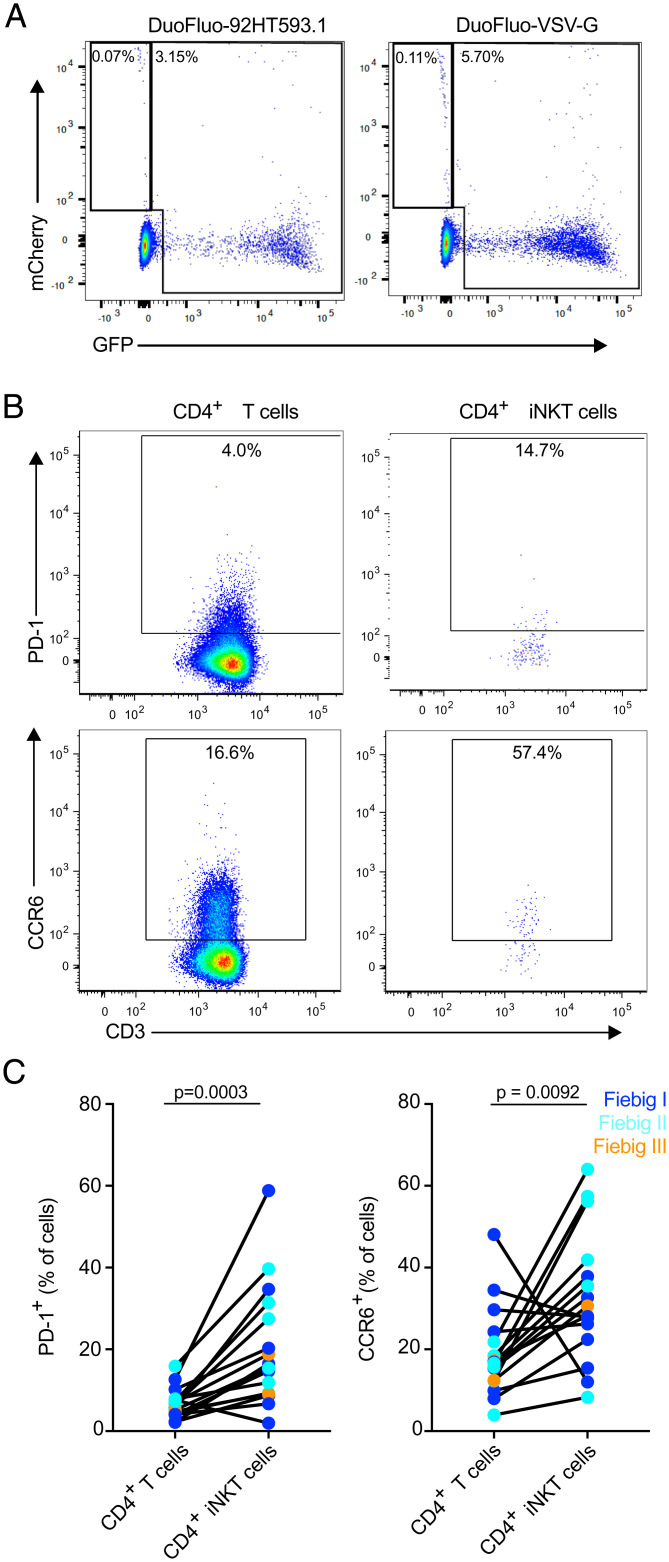
CD4^+^ iNKT cells express markers associated with the HIV reservoir. (*A*) Representative flow plots showing GFP and mCherry expression by iNKT cells 5 d postinfection with HIV-1 DuoFluo. Results are representative of three healthy donors. (*B*) Representative flow plots showing the expression of PD-1 and of CCR6 by peripheral blood and colonic CD4^+^ T cells and CD4^+^ iNKT cells. (*C*) Expression of markers enriched on latently infected cells, PD-1 (*Left*) and CCR6 (*Right*), by peripheral blood CD4^+^ T cells and CD4^+^ iNKT cells from ART-treated, HIV-infected individuals (*n* = 16).

To test the hypothesis that iNKT cells are infected by HIV-1 in vivo, we sorted peripheral blood iNKT cells from AHI from five untreated subjects and measured cell-associated HIV-1 DNA. The median level of cell-associated HIV DNA in iNKT cells was 2.2 copies per 1,000 cells (*SI Appendix*, Fig. S11). The presence of cell-associated spliced (*env* and *tat*/*rev*) and unspliced HIV-1 RNA (*gag* and *LTR*) was measured by qRT-PCR. Both spliced and unspliced HIV-1 RNA were present in iNKT cells at peak VL (*SI Appendix*, Table S3). These results provide direct ex vivo evidence of the productive infection of iNKT cells. To investigate if HIV latency can be established in iNKT cells, we performed in vitro infection of expanded iNKT cells from peripheral blood of HIV-1–uninfected individuals with the DuoFluo HIV-1 construct that allows for the identification of latently infected cells (41). A small population of mCherry^+^ GFP^−^ iNKT cells corresponding to latently infected cells could be identified in all donors tested for both Env- and VSV-G–mediated entry (Fig. 7*A*). As PD-1 and CCR6 have been identified as markers that are enriched in latently HIV-infected cells (42–45), we evaluated their expression on peripheral blood CD4^+^ iNKT cells of ART-treated, HIV-infected individuals (Fig. 7*B*). Peripheral blood CD4^+^ iNKT cells expressed significantly higher levels of PD-1 (*P* = 0.0003) and CCR6 (*P* = 0.0092) compared to conventional CD4^+^ T cells (Fig. 7*C*). Despite the extremely small size of the reservoir in these study participants (46), HIV DNA was detected at low levels in sorted CD4^+^ iNKT cells from 2 out of 10 participants 6 mo after ART initiation (*SI Appendix*, Table S4). These results indicate that iNKT cells express phenotypic markers associated with HIV-1 reservoirs on conventional CD4^+^ T cells and that these cells may contribute to HIV persistence during ART.

## Discussion

In this study, we evaluated the frequency, phenotype, and function of iNKT cells longitudinally during untreated and treated AHI. Both peripheral blood and colonic CD4^+^ iNKT cells decline in AHI, with significant losses observed at the time of peak VL (median day 16 after the first positive HIV RNA test), corresponding to Fiebig stage III. ART initiation during Fiebig stages I and II prevented the reduction of circulating and colonic CD4^+^ iNKT cells, but 2 y of treatment was insufficient to restore the colonic CD4^+^ iNKT cell population once it had been lost. This is in contrast to other immune populations present in the GI tract, such as CD4^+^ CCR5^+^ T cells and Th17 CD4^+^ T cells, that have been shown to be preserved 6 mo after ART initiated during AHI (30). The kinetics of the depletion of colonic CD4^+^ iNKT cells and the lack of complete reconstitution after ART are similar to what we have reported for colonic α4β7^+^ CD4^+^ T cells (47). Furthermore, colonic CD4^+^ iNKT cells undergo a more severe loss than conventional CD4^+^ T cells in AHI, suggesting that they may be preferentially depleted by HIV-1 during early infection. In support of this notion, we found that iNKT cells express higher levels of CCR5 and α4β7, two receptors involved in HIV-1 binding, making them potential targets for HIV-1 infection. Multiple reports have suggested that α4β7^+^ CD4^+^ T cells are preferentially infected by transmitted founder HIV-1 or simian immunodeficiency virus (SIV) (48–51), suggesting that the expression of α4β7 by CD4^+^ iNKT cells may explain their preferential infection and depletion early in infection. Indeed, we and others have reported that the susceptibility of iNKT cell to in vitro infection is higher than that of conventional CD4^+^ T cells (13, 14). Moreover, the frequency of peripheral blood CD4^+^ iNKT cells preinfection correlated with peak viremia. Together, these results suggest that CD4^+^ iNKT cells represent a pool of susceptible cells in which the virus can replicate during the earliest stages of HIV-1 infection. Colonic CD4^+^ iNKT cells levels during AHI were inversely associated with levels of soluble markers of innate activation, suggesting that the depletion of these cells may contribute to inflammation, as previously suggested from studies in chronic infection (22, 25).

Similar to what was previously described in peripheral blood (13), we observed an inverse association between VL and the frequency of colonic CD4^+^ iNKT cells. We also determined the presence of cell-associated, viral DNA and spliced and unspliced viral RNA in sorted peripheral blood iNKT cells, supporting that these cells are infected in vivo. It is possible that the loss of CD4^+^ iNKT cells could be due, at least in part, to HIV-1–induced cytopathicity. Increased *CASP1* and *IFI16* gene expression is compatible with a role for pyroptosis, following the sensing of infection (33, 52, 53). Furthermore, the constitutive expression of PLZF confers an increased propensity to apoptosis in iNKT cells (54), which might further contribute to their depletion in AHI. Coupled with no observable change in chemokine receptor gene expression in iNKT cells in AHI, these data suggest that the reduction of circulating iNKT cells seen in HIV-1 infection may be caused primarily by cell death rather than by a redistribution of the cells to the GI tract. However, we have not directly measured cell death or caspase activation because of sample limitations. The susceptibility of CD4^+^ iNKT cells to HIV-1 infection raises the possibility that these cells may contribute to the viral reservoirs. Long-lived reservoirs are believed to be comprised of latently infected memory CD4^+^ T cells, and the majority of iNKT cells maintain a central memory phenotype (55–57). We have used an in vitro infection model as a proof of principle that iNKT cells can be latently infected by HIV-1. Moreover, CD4^+^ iNKT cells from patients on ART express markers associated with HIV reservoirs on conventional CD4^+^ T cells, such as PD-1 and CCR6. Further work is needed to confirm that the expression of these receptors are also enriched in latently HIV-infected CD4^+^ iNKT cells. Given their relatively low frequency, iNKT cells may contribute to a small proportion of the HIV-1 reservoirs. Future studies are needed to investigate the exact contribution of iNKT cells to the replication competent reservoirs.

The activation of peripheral blood iNKT cells observed in untreated AHI could possibly be attributed to the direct TCR activation of iNKT cells after the recognition of self-lipid antigens induced by viral infection (24, 58) or by indirect cytokine activation (59, 60). However, the activation of iNKT cells at peak viremia was not associated with levels of sCD14, VL, IL-12, or IL-6. Thus, the underlying mechanisms driving their activation remains unclear. It is possible that other cytokines not evaluated here, such as IL-18, are contributing to iNKT cell activation. We observed the decreased capacity for cytokine production by iNKT cells after in vitro stimulation at the early chronic infection time point. At the same time point, TIGIT surface expression in iNKT cells also trended upward, suggesting a possible role for check point inhibitors in iNKT cell exhaustion. The transcription factor GATA3 is known to be important for iNKT cell functionality (36), and we speculate that the decreased expression of *GATA3* in iNKT cells during AHI could contribute to the functional impairment of iNKT cells in early chronic infection. iNKT cells can also have a cytotoxic function (7), but this could not be evaluated because of sample limitations. Interestingly, the initiation of ART in Fiebig stages I or II prevents peripheral blood iNKT cell loss of function. However, 2 y of treatment, initiated during Fiebig stage III, was not sufficient to fully restore colonic CD4^+^ iNKT cells. It is possible that a longer time on treatment may restore this population. Immunotherapies such as IL-15, known to enhance survival of iNKT cells (61) and also reactivate HIV-1 (62), could possibly be combined with ART to both help restore this population and reduce the reservoir. Overall, we have shown that ART initiated in Fiebig stages I or II can preserve peripheral blood iNKT cells but that colonic CD4^+^ iNKT cells are preferentially lost during AHI and do not recover to normal levels after ART initiation during Fiebig stage III.

## Materials and Methods

### Study Participants.

Information on the RV217, RV254, and RV304 study participants is available in the online supplementary material. The RV217 ECHO study has been described previously (32). Briefly, the RV217 study enrolled consenting adults from key populations at four clinical research sites in Kenya, Uganda, Tanzania, and Thailand. HIV-uninfected participants were screened twice weekly for HIV-1 infection by finger pricks and nucleic acid amplification testing (NAAT; Aptima HIV-1 RNA qualitative test, Hologic Inc.). Enrollees with reactive NAAT were enrolled in a second phase of the study that included the intensive sampling of larger blood volumes throughout acute infection and into chronic infection. The individuals were monitored longitudinally over time twice weekly for VL, and rising and declining VLs allowed for the identification of a peak inflection point (32). All HIV-1–positive participants were referred to care providers for management of the infection based on national guidelines. Treatment was usually available at no cost through host nation care and treatment programs. The cases presented in this study include a selected set from a group of 20 RV217 participants for whom cryopreserved PBMC were available prior to infection and at least three postinfection time points corresponding to peak VL (median days since first positive test for HIV-1 RNA = 16), set point VL (median days since first positive test for HIV-1 RNA = 43), and early chronic infection (median days since first positive test for HIV-1 RNA = 85). Lymphocyte absolute counts were performed real time on whole blood using the Trucount, lyse no wash, Multitest platform (Becton Dickinson Biosciences) to enumerate T cell, B cell, and NK cell subsets.

The RV254/SEARCH 010 study is an ongoing acute infection cohort based in Bangkok, Thailand (clinicaltrials.gov identification NCT00796146). ART was provided within a few days of diagnosis under a separate protocol (clinicaltrials.org identification NCT00796263). Blood samples were screened in real time by pooled NAAT and sequential EIA, according to published methods (63). Participants who had positive NAAT (confirmed by quantitative HIV-1 RNA) and nonreactive HIV IgG were enrolled in the RV254/SEARCH 010 cohort. The sampling of mucosal biopsies was performed by sigmoidoscopy as an optional study procedure at the time of HIV diagnosis (*n* = 23) and 2 y after ART initiation (*n* = 20). ART was initiated at a median 4 d from cohort enrollment. The first seven participants included in this analysis were treated with standard doses of tenofovir/emtricitabine/efavirenz/raltegravir/maraviroc, while subsequent participants were randomized to either this regimen or tenofovir/emtricitabine/efavirenz. Plasma, PBMC, and mucosal mononuclear cells (MMC) from HIV-uninfected Thai individuals participating in protocol RV304 (clinicaltrials.gov NCT01397669), who underwent the same procedures, were used as controls.

### Study Approval.

The RV254/SEARCH 010 and RV304/SEARCH 013 studies (clinicaltrials.gov NCT00796146 and NCT01397669, respectively) were approved by the Institutional Review Boards (IRBs) of Chulalongkorn University in Thailand and the Walter Reed Army Institute of Research in the United States. The initiation of ART was voluntary under an accompanying protocol (clinicaltrials.gov NCT00796263) approved by Chulalongkorn University IRB. The RV217 study was approved by the Walter Reed Army Institute of Research in the United States and relevant IRBs in Kenya, Uganda, Tanzania, and Thailand. For all studies, participants gave written informed consent.

### Biopsy Processing and Calculation of the Absolute Number of Colonic T Cell Subset.

Participants underwent a routine sigmoidoscopy procedure under moderate conscious sedation. A total of ∼30 endoscopic biopsies were randomly collected from the sigmoid colon using Radial Jaw 3 biopsy forceps (Boston Scientific), not accounting the visual control for the potential collection of lymphoid aggregates with 20 to 25 processed for flow cytometry analysis within 30 min of collection, as previously described (30). The cell count for all mucosal samples was done manually by Trypan Blue exclusion, which allows the exclusion of epithelial cells because of their morphology compared to lymphocytes. Absolute numbers of CD4^+^ and iNKT cells per gram of gut tissue were calculated by multiplying the total viable lymphocyte count by frequencies of cell subsets obtained from flow cytometric analysis. The total lymphocyte count per gram of tissue was calculated by dividing the viable lymphocyte count by the tissue weight. This proportion was then multiplied by the percent of cells in the live lymphocyte gate and that number was subsequently multiplied by the percent of CD3^+^ lymphocytes. The absolute number of colonic CD3^+^ T cells was used in conjunction with the subset percentages to determine the absolute number of each T cell subset per gram of biopsy tissue.

### Flow Cytometry.

The frequency and phenotype of peripheral blood and mucosal iNKT cells were determined as previously described (64). Briefly, thawed samples were washed, stained with LIVE/DEAD Fixable Aqua Dead Cell dye (Thermo Fisher Scientific), blocked for Fc receptors using normal mouse serum (Thermo Fisher Scientific), and surface stained with antibody mixture. Samples were surface stained at room temperature for 30 min. For panels including CCR5 antibodies, surface staining was performed at 37 °C. Samples were fixed in 2% paraformaldehyde before acquisition on a five-laser, 16-parameter BD LSRII SORP flow cytometer or a four-laser custom-built LSR Fortessa (BD Biosciences). Total evens acquired in the iNKT cell gate ranged from 147 to < 7,000. Other samples used for sorting for downstream transcriptomics were resuspended in sorting buffer (phosphate buffered saline [PBS] containing 1% bovine serum albumin [BSA]) and sorted for bulk iNKT cells for targeted transcriptomics with Fluidigm Biomark. Data were analyzed with FlowJo version 9.9.4 or higher (TreeStar). See *SI Appendix*, *Methods* for specific antibodies used throughout the study. Anti-human α4β7 [clone Act-1, NIH AIDS Reagent Program, Division of AIDS, NIAID, NIH (catalog No. 11718) from Dr. A. A. Ansari (65)] was labeled using Alexa Fluor 647 antibody–labeling kit from Invitrogen.

### Functional Assays.

Functional assays were performed as previously described (66). PBMCs were rested overnight at 37 °C and stimulated with PMA and Ionomycin, as per the manufacturer’s recommendation (eBioscience Cell Stimulation Mixture [500×], Thermo Fisher Scientific) for 6 h. Monensin (eBioscience) and Brefeldin A (BD Biosciences) were added during the stimulation.

### Statistical Analysis.

All statistical analysis was performed using Graph Pad Prism version 8.2.0 for Mac OS (GraphPad Software). Longitudinal comparisons were performed using the Friedman test. Comparisons between HIV uninfected and HIV infected were performed using the Mann–Whitney *U* test. Associations were evaluated using Spearman’s rank correlation. *P* values < 0.05 were considered statistically significant.

## Data Availability

All study data are included in the article and/or supporting information.
